# Integrative Understanding of Familial Impulsivity, Early Adversity and Suicide Risk

**DOI:** 10.3389/fpsyg.2017.02240

**Published:** 2017-12-22

**Authors:** Isabela M. M. Lima, Leandro F. Malloy-Diniz, Débora M. de Miranda, Antônio G. Da Silva, Fernando S. Neves, Sheri L. Johnson

**Affiliations:** ^1^Graduate Program in Molecular Medicine, Federal University of Minas Gerais, Belo Horizonte, Brazil; ^2^Brazilian Association of Psychiatry, Rio de Janeiro, Brazil; ^3^Department of Mental Health, Faculty of Medicine, Federal University of Minas Gerais, Belo Horizonte, Brazil; ^4^Department of Psychology, University of California, Berkeley, Berkeley, CA, United States

**Keywords:** bipolar, impulsive behavior, suicide, attempted, relatives, child maltreatment

## Abstract

**Introduction:** Impulsivity is a core characteristic of bipolar disorder and it was observed as elevated in individuals with the disorder and in their relatives. Both impulsivity and history of maltreatment are risk factors for suicide attempts, however, these two key variables may not be independent, given the fact that parental impulsivity and associated social context could increase the risk of child maltreatment. In this study it was examined the association between the impulsivity of relatives and child maltreatment taking into consideration the conjoint and unique effects of these two variables on the risk of suicide attempts among the patients.

**Materials and Methods:** Participants of the study consisted of 117 patients diagnosed with bipolar disorder and 25 first-degree relatives. Linear regression model was conducted to describe associations between facets of impulsivity of relatives and levels of child maltreatment reported by patients. The independent associations of suicide attempt history with the dimensions of impulsivity of the patient and maltreatment were tested by multinomial logistic regression.

**Results:** Impulsivity of relatives and, more specifically, inhibitory control can predict the maltreatment of the patient. Inhibitory control and emotional abuse were related, conjointly, to a greater likelihood of having a history of more than one suicide attempt.

**Discussion:** Considering that the impulsivity of relatives predicts child maltreatment, it is possible that a genetically shared impulsivity is an underlying feature associated with the history of multiple suicide attempts. These findings highlight the importance of considering child maltreatment, impulsivity and suicide attempt history in integrative models.

## Introduction

Bipolar bisorder (BD) consists of a heterogenic group of mood pathologies, which varies in terms of amount and severity of depressive and/or manic episodes, presence of psychotic symptoms, comorbid diagnostics, number of hospitalizations and suicide risk.

There are at least two main BD subtypes. *Bipolar I* disorder is characterized by the presence of at least one manic episode. Mania, in turn, is characterized by a period of at least 1 week of elevated, expansive or irritable mood, increased energy, and more talkativeness and engagement in risky activities (American Psychiatric Association [APA], 2013). *Bipolar II* is described as the presence of at least one previous major depressive episode for at least 2 weeks and one hypomanic episode that lasts a minimum of 4 days (American Psychiatric Association [APA], 2013).

Approximately one quarter of patients with *Bipolar I* report suicide attempts (Merikangas et al., 2011) and suicide accounts for 10 and 6.7% of deaths in *Bipolar I* and *II*, respectively (Angst et al., 2013). Hence, BD presents the highest absolute risk of suicide among different psychiatric conditions (Nordentoft et al., 2011). The focus of this paper is to understand the conjoint effects of two key factors for suicidality within BD: child maltreatment and impulsivity. With the aim to shed light into how impulsivity, maltreatment and suicidality interact, the goal of this study is to develop and test a more integrative model of these variables.

Impulsivity is considered to be a core feature of BD (Swann et al., 2003). To begin with, the diagnostic criteria for mania involves impulsivity. Even after manic episodes clear out, however, a growing body of research suggests that high levels of impulsivity persist (Swann et al., 2003). It is also noteworthy that impulsive behaviors can be transmitted through generations since it is observed to be elevated even in family members who do not meet diagnostic criteria for BD (Fortgang et al., 2016). According to the Self-Regulation Intergenerational Transmission Model, the diminished self-regulation that contributes to impulsive behavior is multifactorial, involving prenatal, social mechanisms of transmission, such as marital and parent-child interaction, as well as neurobiological mechanisms (Bridgett et al., 2015). Impulsivity is associated with low quality of life (Victor et al., 2011) and dysfunctional behaviors such as aggressiveness within BD (Johnson and Carver, 2016).

Particularly, impulsivity seems to be related to suicidality within BD (Johnson et al., 2016). Impulsive behavior triggered by emotion is related to history of suicidal ideation, self-harm, and suicide attempt in BD (Johnson et al., 2016) and also among those without BD (Kasen et al., 2011; Black and Mildred, 2013; Auerbach et al., 2016). These findings converge with the results of a longitudinal study, which indicates that impulsivity may predict suicide attempts over a 15-year period in the general population (Kasen et al., 2011).

A separate literature indicates that early adversity and maltreatment are major concerns within BD. Rates of child abuse and neglect are quite high in those with the disorder, with exposure estimates as high as 51% (Daruy-Filho et al., 2011). Early adversity and maltreatment have been associated with early bipolar onset and also to contribute to a poor prognosis within BD, including more symptom severity, more relapse and lower levels of functioning (Daruy-Filho et al., 2011). Child maltreatment is also a predictor of the risk of suicide within BD (Norman et al., 2012). Indeed, a wide range of forms of child maltreatment, including sexual abuse, emotional abuse, physical abuse and emotional neglect, have been individually associated with the risk of attempted suicide within BD (Norman et al., 2012).

Although both impulsivity and childhood adversity seem to be important in understanding bipolar suicidality, these two variables have been studied separately in the literature on BD. However, it is possible that impulsivity among family members may increase the risks of exposure to childhood adversity in offspring.

Considering this background, it was hypothesized that patients whose relatives have high levels of impulsivity may be in a social context that could increase the risk of abuse. First, the degree of overlap of early adversity and impulsivity among individuals with BD was assessed, then the conjoint and unique effects of early adversity and patient impulsivity as correlates of suicide attempt were considered. That is, it was examined: (1) whether the impulsivity of relatives can predict the levels of child maltreatment; (2) the extent to which the impulsivity of the patients and maltreatment dimensions are associated with suicide attempts.

## Materials and Methods

### Sample

The sample consisted of a group of 117 bipolar patients and 25 first-degree relatives. The inclusion criteria taken were being aged between 18 and 60 years. Exclusion criteria were based on neurological conditions. The bipolar group consisted of individuals who had been previously diagnosed with BD by a psychiatrist and were being treated in a bipolar outpatient clinic which is part of Brazilian Public Health System. The bipolar diagnosis was confirmed through the administration of the MINI Plus 5.0 diagnostic interview (Sheehan et al., 1997). Each patient was asked to indicate a first-degree adult relative to compose the sample of relatives. However, only part of them attended to the scheduled appointment. Only a few of them met the inclusion criteria, resulting in a small sample of 25 relatives. Inclusion and exclusion criteria were the same applied to patients with the addition that they could not present psychiatric diagnosis, assessed by the administration of MINI Plus 5.0 diagnostic interview (Sheehan et al., 1997). First degree relative was considered as being parents’ siblings or children of patients. No more than one relative per patient was assessed. Fifteen of the 25 relatives assessed were parents of patients, on the majority being their mothers. The Ethics Committee of the Federal University of Minas Gerais approved this study (N° 064/09) and all of the participants filled a written informed consent procedures before taking part in the study procedures.

### Measures

Interviews and instruments were administered to patients in order to assess impulsivity, child maltreatment, suicide attempt history and mood states. Family members completed parallel measures of their personal levels of impulsivity and mood states.

#### Barrat Impulsiveness Scale – BIS – 11 (Patton et al., 1995)

The BIS – 11 is a 30-item self-rated scale designed to assess impulsive thought and behavior. The Brazilian version of the scale was used, which has already been validated (Malloy-Diniz et al., 2010, 2015). Previous analysis identified two factors from the extracted of the Brazilian version of the scale: Inhibition control (α = 0.79), defined as the ability to inhibit a proponent behavior and attentional control, and Non-planning (α = 0.62), defined as the decision making that requires cost-benefit evaluation between short and long term consequences within an emotional context (Malloy-Diniz et al., 2015).

#### Childhood Trauma Questionnaire – CTQ (Bernstein et al., 2003)

The CTQ is a 28-item self-report questionnaire of frequency of traumas experienced before the age of 12, in 5-point likert scale, ranging from *never* to *always*. The Brazilian version of this scale was taken into consideration, which presents five subscales: emotional (α = 0.88), physical (α = 0.92), and sexual abuse (α = 0.97), as well as emotional (α = 0.94) and physical neglect (α = 0.66), (Grassi-Oliveira et al., 2006, 2014).

Suicide attempt history Suicide attempt history was assessed through a self-report question that classified patients as those who never attempted suicide, the ones that attempted once or those who attempted more than once. The question was “Have you ever attempted suicide?” The patient was asked to mark one of three options: Never attempted suicide, attempted once, attempted more than once. In the end, suicide attempt was defined as an act with clear intention to kill oneself.

#### Beck Depression Inventory – BDI (Beck, 1961)

The BDI is one of the most commonly used self-rated depression scales. The 21-item cover depressive symptoms such as energy level, hopelessness, suicide ideation and self-punishment. Each item is rated on a scale of 0–3 and higher scores reflect greater depression severity. The Brazilian version shows internal consistency of 0.81 (Gorenstein and Andrade, 1996).

#### Young Mania Rating Scale – YMRS (Young et al., 1978)

The YMRS scale is a semi-structured interview and rating system used to quantify severity of mania symptoms observed during clinical interview and in the past 48 h. It consists of 11 questions that asses mood, activity level, psychomotor agitation, sexual interest, appearance, irritability, insight, sleep, aggressiveness and content of thoughts. Each item is scored on a scale of 0–4, with the exception of items assessing irritability, speech, content of thoughts and disruptive behavior. These have double the scoring weight and are scored from 0 to 8. Maximum score possible is 56 points. A trained clinician who had at least 2 years of experience in a bipolar outpatient clinic completed the interview and ratings. The Brazilian version of YMRS was used, which presented intra-class correlation coefficient of 0.80 and internal consistency of 0.67 (Vilela et al., 2005).

### Statistical Analysis

All of the statistical analysis were performed in SPSS software, version 21.0. The following methods were selected to describe the associations of (1) impulsivity of relatives and levels of child maltreatment of patient; and (2) impulsivity and maltreatment dimensions of patient with history of suicide attempts and its reoccurrence. Since the sample size was unbalanced for relatives and patients, the analysis were divided in those two steps. First, principal components factor was conducted, using the oblimin rotation on the child maltreatment items of all 117 patients, in order to determine the number of statistically independent dimensions of trauma and reduce subsequent dependent variables in subsequent linear regression model. This was considered a preliminar requirement for step one, since the sample size was not enough to allow consideration of all dimensions of maltreatment as a dependent variable in further regression.

To examine the effect of impulsivity of relatives scores on child maltreatment of patients, a linear regression model was conducted with both BIS factors as independent variables. This step considered 25 patients and their respective relatives.

As a second step, a multinomial logistic regression model was computed to assess whether maltreatment and impulsivity predicted suicide attempt history among the 117 individuals with BD. The targeted categories were *No history of suicide attempt; One previous suicide attempt;* and *More than one previous suicide attempt.*

Depression and mania ratings were considered as potential confounds and were controlled as needed in both steps.

## Results

The demographic characteristics of the sample, as well as levels of impulsivity and child maltreatment, are described in **Table 1**. Approximately 67% of the patients met diagnostic criteria for *Bipolar I* and 32.3% for *Bipolar II* disorder.

**Table 1:** Sample characteristics.

	Patients (*N* = 117)		Relatives (*N* = 25)	
	*N*	%	*N*	%
**Level of education**
Secondary education or below	69	58.9	15	60.0
Partial undergraduat	14	12.0	4	16
Undergraduate	29	24.8	5	20.0
Graduate degree	5	4.3	1	4
Gender – Women	78	66.7	17	68.0
**Suicide attempt history**
No history of suicide attempt	81	69.2	–	–
One previous suicide attempt	20	17.1	–	–
More than one previous suicide attempt	16	12.7	–	–

	**Mean**	***SD***	**Mean**	**SD**

Age	43.60	12.16	41.89	15.75
Beck Depression Inventory	14.34	11.29	11.32	10.67
Young Mania Rating Scale	1.90	3.18	3.09	3.86
BIS – Inhibition control	40.55	9.14	35.52	6.96
BIS – Non-planning	23.87	6.01	25.28	4.54
Physical neglect (3 items)	4.60	1.96	–	–
Emotional neglect (7 items)	13.87	6.12	–	–
Physical abuse (5 items)	7.99	3.92	–	–
Emotional abuse (5 items)	10.32	4.99	–	–
Sexual abuse (5 items)	6.85	3.80	–	–

### Principal Components Analysis of the Childhood Abuse Scale

Principal component analysis are shown in **Table 2**. The results showed no multicollinearity and the sample size was in accordance to Kaiser-Meyer-Olkin measure of sample adequacy (KMO = 0.71), and KMO for individual variables were equal or higher than 0.67. Sphericity as tested by Bartlett’s test indicated that the correlations between variables were adequate for Principal Components Analysis (*X*^2^ = 177.41, *df* = 10, *p* < 0.001). One factor surpassed Kaiser’s criterion of Eigenvalue higher than 1 and explained 52.58% of the variance. Considering that the statistical requirements to consider maltreatment as an unique factor were met, maltreatment was taken as one factor on analysis regarding associations between impulsivity of relatives and maltreatment. These results show that the frequency of specific types of maltreatment are not dissociated from others. That is, those patients who tend to experience elevated levels of one type of maltreatment may also have higher probability to experience other forms of it.

**Table 2:** Factor loading of Childhood Trauma Questionnaire subscales

Subscale	Factor 1: Maltreatment
Emotional neglect	0.877
Physical neglect	0.736
Emotional abuse	0.802
Psysical abuse	0.699
Sexual abuse	0.431

*Extraction Method: Principal Component Analysis. Rotation Method: Oblimin with Kaiser Normalization*.

### Impulsivity of Relatives Levels Correlate with Levels of Childhood Maltreatment of Patients

Before conducting the analysis of the impulsivity effects, potential confounds of manic (YMRS) and depressive (BDI) symptoms were considered by testing correlations with maltreatment of patient scores. Neither the YMRS, *r* (DF) = -0.07 (24), *p* = 0.74 nor the BDI, *r* (DF) = 0.31 (24), *p* = 0.13 were correlated with maltreatment. More specifically (as shown in **Table 3**), results of the linear regression show that first-degree BIS Inhibitory Control scores of relatives correlate significantly with patient report of General maltreatment, although Non-planning scores did not contribute to additional variance. Impulsivity of relatives explains a considerable proportion of the variance, *r*^2^ = 0.30, *N* = 25, *p* < 0.05. Therefore, relatives impulsivity of relatives accounts for 30 percent of variance in self reported levels of maltreatment, showing that inhibitory control in relatives seem to impact on frequency of perceived maltreatment of patients.

**Table 3:** Linear regression of Family Impulsivity Scores as Predictors of Maltreatment of Patients Scores (*N* = 25).

	*B*	*SE* B	b
Constant	-0.10	1.12	
BIS – Inhibitory Control	0.05	0.02	0.43^∗^
BIS – Non-Planning	-0.06	0.03	-0.36

*r^*2*^ = 0.30, *N* = 25, ^∗^*p* < 0.05*.

### Maltreatment of Patients and Impulsivity Predict the Number of Previous Suicide Attempt

As shown in **Table 4**, multinomial logistic regression was performed with BIS and Maltreatment scores as predictors of suicide attempt (never, once, more than once) as an outcome variable. Diminished inhibitory control was robustly correlated with number of suicide attempts, distinguishing specifically those who attempted suicide once or more from those who never attempted.

**Table 4:** Multinomial logistic regression for history of suicide attempt.

		95% CI for Odds Ratio		
	B (*SE*)	*Lower*	Odds Ratio	*Upper*
**No history of SA vs. more than one SA**				
Intercept	7.56 (2.81)			
BIS – Inhibition control	-0.11 (0.04)*	0.83	0.89	0.97
BIS – Non-planning	0.01 (0.06)	0.90	1.01	1.13
Physical neglect	0.21 (0.23)	0.79	1.23	1.93
Emotional neglect	-0.14 (0.08)	0.74	0.87	1.02
Sexual abuse	-0.07 (0.08)	0.80	0.93	1.09
Physical abuse	-0.14 (0.09)	0.73	0.87	1.04
Emotional abuse	0.14 (0.09)	0.96	1.15	1.38
**One previous SA vs. more than one SA**				
Intercept	6.71 (3.34)			
BIS – Inhibition control	-0.15 (0.05)*	0.78	0.86	0.95
BIS – Non-planning	-0.09 (0.08)	0.79	0.92	1.07
Physical neglect	0.23 (0.25)	0.77	1.26	2.08
Emotional neglect	-0.08 (0.09)	0.77	0.92	1.11
Sexual abuse	0.02 (0.08)	0.87	1.02	1.20
Physical abuse	-0.11 (0.11)	0.73	0.89	1.10
Emotional abuse	0.27 (0.11)*	1.06	1.31	1.62

*R^*2*^= 0.29 (Cox and Snell), 0.36 (Nagelkerke). Model *x*^2^ = 39.99, *p <* 0.001. *df = 14, ^∗^p* < 0.05*.

Child Maltreatment scores were considered separately to provide richer information regarding factors associated with presence and/or recurrence of suicide attempt. Only the Emotional abuse factor was significantly related to suicidality and it was only significant in differentiating repeated attempters from those with only one attempt. The correlations between impulsivity and maltreatment dimensions are shown in **Table 5**.

**Table 5:** Spearman’s rho correlations between BIS-11 and CTQ patient’s subscale scores.

	1	2	3	4	5	6
(1) BIS – Inhibition control	–	–	–	–	–	–
(2) BIS – Non-planning	-0.35^∗∗^	–	–	–	–	–
(3) Physical neglect	0.10	-0.14	–	–	–	–
(4) Emotional neglect	0.15	-0.21^∗^	0.64^∗∗^	–	–	–
(5) Sexual abuse	0.18	-0.02	0.18	0.20^∗^	–	–
(6) Physical Abuse	0.13	-0.00	0.36^∗∗^	0.45^∗∗^	0.23^∗^	–
(7) Emotional Abuse	0.36^∗∗^	-0.09	0.30^∗∗^	0.58^∗∗^	0.40^∗∗^	0.51^∗∗^

^∗^*p < 0.05; ^∗∗^*p* < 0.001*.

## Discussion

Many studies indicate that impulsivity and early adversity experiences are both correlated with suicidality within BD. Despite evidence that familial impulsivity may feature a context prone to greater risk of abuse, researchers have tended to consider these two variables separately. The goal of this study was to develop a more integrative understanding of familial impulsivity and early adversity, and also to consider whether patient’s impulsivity and abuse contributed independently to the risk of suicide attempts for individuals with BD. Since those that repeatedly attempted suicide may represent a subset of more severe patients, beyond discrimination of the presence of suicide attempt history, it was also an aim to describe aspects that could account for reincidence of suicide attempt.

Our findings confirmed that problems of patients with inhibitory control were tied to suicidality. Furthermore, findings extended previous work outside the BD diagnosis by showing that poor inhibitory control in relatives was related to the frequency of child maltreatment experienced by patients. Although these findings do not test direct influences of impulsivity of relatives and risk of suicide attempt in patients, it highlights that emotional abuse is related to a greater risk of recurrent suicide attempts even after accounting for deficits in inhibitory control in patients. These novel findings provide initial data to foster investigations on further unified and transgenerational perspective on impulsivity, adversity and suicidality.

This study, such as previous ones, suggests that the effects of impulsivity on suicidality within BD do not seem to generalize across measures of impulsivity, which indicates that it must be considered a multifaceted construct (Malloy-Diniz et al., 2009, 2011; Watkins and Meyer, 2013; Johnson et al., 2016). Previous works have found that impulsivity in the context of emotion is particularly predictive of suicidality, moreover this form of impulsivity would be helpful to be considered in future models of links between early adversity, impulsivity and bipolar suicidality.

The findings of this study dovetail with other researches that increasingly documents the complex interplay between maltreatment and impulsivity. Child maltreatment may not be independent of impulsivity of relatives. However, the results do not show the underlying causes that link them. Within BD, relatively few relatives meet full diagnostic criteria for the disorder, but many experience high levels of impulsivity when compared to healthy controls (Hıdıroğlu et al., 2013). One possibility is that lack of inhibitory control in relatives is a marker of familial contexts which are prone to maltreatment behaviors. Higher familial impulsivity may lead to a more chaotic family environment that allows these other adversities to unfold. History of maltreatment, in turn, influence cognitive and emotional development, which intensify the risks of impulsivity and poor constraint over harmful behaviors (Braquehais et al., 2010; Daray et al., 2016). Inhibitory control shared by patients and relatives as an impulsivity dimension fosters dysfunctional behaviors such as maltreatment and suicide behavior. The reasons for relatives and patients to present dysfunctional may be diverse, possibly involving learnt behavior, developmental interferences of one behavior over another as well as shared genetic aspects that this study could not assess. That is, above and beyond the genetic influences, maltreatment has been shown to predict impulsivity over time (Braquehais et al., 2010; Daray et al., 2016).

Current findings indicated that inhibitory control and emotional abuse set the stage for recurrent suicide attempts rather than single attempts. The number of suicide attempts is a particularly robust predictor of suicide completion (Watkins and Meyer, 2013). Those that attempted suicide repeatedly also tend to present more severe dysfunctionality, as evidenced by their higher numbers of hospitalizations. Therefore, the clarification of inhibitory control as an specific aspect related to this subset of patients may be a promising target for treatments that aim to reduce recurrent suicide behavior and its dysfunctional consequences. If replicated, the current findings highlight the importance of considering specific types of early abuse as serious markers of more pronounced suicide risk.

Despite the potential clinical importance of these findings, the study is limited by the reliance on self-report measures of impulsivity and maltreatment, and also by the small number of relatives assessed. It is also important to consider that siblings and children were included in sample of relatives. Therefore, since it is less plausible that their impulsivity leads to maltreatment, it is not possible to conclude that the impulsivity observed in relatives leads to maltreatment. Instead, it may be more plausible to consider that impulsivity observed in relatives can be considered a family vulnerability marker to impulsive behaviors such as maltreatment. The small sample size precluded the ability to examine more refined dimensions of suicide attempts, such as lethality and age of onset. Furthermore, medication or comorbidities were not controlled. Despite the limitations, this study supports the relevance to develop approaches encompassing the transgenerational perspective on impulsivity and maltreatment in relation to suicide attempts in BD.

Current findings, if replicated, would have important clinical implications. First, emotional abuse and inhibitory control were independently associated with number of suicide attempt so they both represent important potential treatment targets. Second, interventions to address impulsivity may need to consider that this personality trait evolved in a family context. One possible approach for those who are seeking family therapy might be to integrate a consideration of family levels of impulsivity into that work. Impulsive behaviors of patients and family members could be addressed by teaching self-regulatory strategies, but also by considering communication effects and strategies in contexts in which multiple members of a family may have these concerns. By doing so, it may reduce the risk of high levels of conflict and potentially provide some protection from emotional abuse. As a whole, it is expected that these preliminary findings provide insight into some important links between variables that have been studied separately in understanding suicidality within bipolar disorder. Future studies should investigate causal relationships using larger samples and longitudinal designs.

## Ethics Statement

This study was carried out in accordance with the recommendations of the Ethics Committee of the Federal University of Minas Gerais with written informed consent from all subjects. All subjects gave written informed consent in accordance with the Declaration of Helsinki. The protocol was approved by the Ethics Committee of the Federal University of Minas Gerais.

## Author Contributions

IL: Contributed on writing, performing statistics and delineating method. LM-D: Contributed on discussion, data collection and writing. DdM: Contributed on writing, discussion and data collection. ADS: Contributed on writing and reviewing. FN: Contributed on data collection, method and on delineating the study designed. SJ: Contributed on delineating, methods, statistics, discussion and writing.

## Conflict of Interest Statement

The authors declare that the research was conducted in the absence of any commercial or financial relationships that could be construed as a potential conflict of interest.
